# The power that comes from within: female leaders of Rio de Janeiro’s *favelas* in times of pandemic

**DOI:** 10.1177/1757975921994690

**Published:** 2021-06

**Authors:** Nilza Rogeria De Andrade Nunes

**Affiliations:** Pontificia Universidade Catolica do Rio de Janeiro, Rio de Janeiro, Brazil

**Keywords:** women, favela, community-based organization, COVID-19, Brazil

## Abstract

This paper aims to present how the female leaders of the *favelas* of Rio de Janeiro/Brazil have been protagonists in coping with the demands arising from COVID-19. The city has approximately 2 million residents living in 763 *favelas*. There is no strategic planning on the part of the government with coordinated actions related to the specificities of these territories — producing an escalation of demands due to the living and health conditions of the residents. It is in this multifaceted reality, with urgencies and emergencies, that we highlight the role of community by strengthening the local support networks that are built like webs inside the *favela* and beyond. Our statement is based on a qualitative study involving 111 such women, distributed across 105 *favelas*. Correlating their practices, 97% say they support health promotion through the strengthening of popular participation towards community development and defense of rights, and mobilization of health services to meet these populations’ needs, among other actions. With the presence of public agents in these places restricted in times of pandemic, these women often take up the duties of the local authorities to ensure food security, good communication among local residents on health standards, hygiene measures, assistance to the most vulnerable, etc. Perceived by community members as replacing the role of government agencies, they develop a particular way of doing politics. Calling upon resistance and solidarity, they transform this micro-power into effective changes to cope with the inequities and in benefit of citizenship and the other residents of the *favelas* where they live.

## Introduction

The COVID-19 pandemic affects the rich and the poor, but its brutal aggression and spread finds much more fertile ground when it affects groups that are in more precarious conditions of life and health. Social isolation as a coronavirus control strategy — for those, the street is the only space beyond their houses; hand washing and personal hygiene for those who do not have regular access to water; protecting hands, mouth, nose and eyes, for those who do not have any source of income or dignified means of survival; among many other weaknesses — exposes the inequities to which the residents of *favelas* and peripheral areas of Rio de Janeiro are subjected.

It is in this context that it is shown how women in the *favelas* are responding to this as leaders, the purpose of this paper being to document their practices and attitudes in coping with the pandemic. Being a woman, being black, and living in a *favela* in Rio de Janeiro is to be subject to triple discrimination, since the stereotypes generated by sexism, racism, and social status put such women in a position of inferiority. Thus, we see a confluence of oppressions that fall upon the women who live in the *favelas*. The factors of gender, race, and class that lead us to the notion of intersectionality, a term coined by Kimberlé Crenshaw (1), are addressed in this paper.

Based on certain theorists of black feminism (2–7), it can be affirmed how structural oppressions are interconnected in a domination matrix that influences all levels of social relations permeating individual and collective plans — and how these structures are visible and permeable when women from *favelas* are referred to. However, we also show that some of them subvert this order and push the limits of the envelope imposed by the current social structure.

Women, popularly appointed as community leaders or social activists, have been building and consolidating their place of speech (8) and public recognition across diverse agendas since the 1980s — gaining momentum and visibility in the 1990s. They subvert the order that has, historically, made them invisible, and developed their own particular way of conducting politics.

The protagonists here on the scene are geopolitically located in the socio-spatially segregated territories that are called *favelas* in Rio de Janeiro — permeating the urban fabric of the city of Rio de Janeiro, these ‘ghettos’ are constituted and expressed in a mosaic of violence and rights violations. Due to their negative image, synonymous with insecurity and violence, their existence has historically been denied. But despite all this, they play an essential geographical, economic, social, and political (9–11) role in the city.

This homogenizing view of the *favela*, associated with a discourse aimed almost exclusively at what is lacking, coincides with the constructed concept of not recognizing the *favela* resident as an active agent, inserted within the time and space of the city, and thus a citizen with rights. Defined by physical and symbolic borders, they form areas of separation and contact for socio-spatial practices that are drawn in the landscape. They mark and individualize places and forms of belonging and appropriation of the urban space (10).

Even though these places are strongly demarcated by visible borders, women have been affirming their existence and showing resistance. They manage territorial networks (12–14) that are built like webs within and beyond the *favela* territory and they are engaged in the search for transformation of a collective that transcends their personal relationships, but calls out to everyone within and to all those around them.

In the exercising of this local power, they push against the limits demarcated by colonialism (15–17) and racism (7,18). We are talking about poor black women who carry in their bodies multiple expressions of a society marked by oppression, patriarchy, and inequality (19). Alongside this, we incorporate white women who are equally poor inhabitants of popular locations and are also subjugated under such conditions of oppression. Thus, our reflections are anchored mainly in the references to black feminism (2,20,21), decolonial studies (15,16,22), and the concept of *favela* as a geopolitical space (9,23,24) — demarcated not only by a sociability that is established within the contradictions between unmet needs and violence, but also by the presence that is established through solidarity and neighborhood ties.

The recommendations made by the World Health Organization (WHO) can hardly be adopted by this huge population contingent, considering the conditions in which they live. How does one protect one’s own health and that of one’s family when one needs to put food on the table every single day? There is a considerable number of unemployed, informal workers, small traders, housekeepers earning by the hour — all involved with job and income-generation activities that are crucial to the survival of these communities, whose vulnerability has been sharply intensified by the pandemic.

Clearly, the pandemic has exposed Brazil as an extremely unequal country with an inadequate social protection system (25–27) — the worsening of the economic crisis has punished the poorest much more and the COVID-19 outbreak and its mortality rate finds more favorable conditions for transmission in the agglomerations within *favelas*.

The lack of public policies related to infrastructure, such as housing, basic sanitation, digital access, among others, explains the worsening of the situation for people who are deemed less important, especially if one considers that it is the poor and blacks who mostly inhabit popular spaces. The public authorities did not develop a specific contingency plan for the *favelas* to face the pandemic — which can be considered another form of violence perpetrated by the State.

The physical distancing during the pandemic is a challenge in *favelas*, since the use of the street as a shared space for meeting and leisure becomes an extension of the home, most of which are very small — with six to nine people per square meter. This is where workers are found, who, when not unemployed or underemployed, work in essential services so that the middle and upper classes can isolate themselves and carry out their activities in the comfort of their privileged locations.

Poor women, who are mostly black or self-declared as black, face unemployment or immersion in the risk of contamination, since Brazilian women are the ones who are most active in the informal work sector and in activities related to the act of caring. Interestingly, the first lethal victim of COVID-19 in Rio de Janeiro was a 63-year-old domestic worker infected in the employer’s home. This detail is symbolic — as it exposes the structural racism (28) perpetrated against women. Considering these initial reflections and the challenges arising from them, we focus on women from *favelas* (29), known leaders recognized for their local activism. The day-to-day construct of this woman and her place of social and political prominence is shaped by practices and attitudes revealing that there is a power that derives from these popular spaces, exercised by women, that involves unique experiences of exclusion, but does not abandon the struggle.

## Locating these women

When we relate the performance of these women to a force that emerges from within the *favela*, we speak of it as the ‘multiplicity of power relationships that are immanent to the domain in which they perform and are constitutive of their organization (30)’. Thus, we understand power as multidimensional and this stance encourages a look at everyday connections and power relations on all scales.

The knowledge of these women is grounded in their daily experiences and this is how they acquire learning — confirming the need to recognize that popular culture comes from the knowledge of the people (31). The protagonism these women perform is focused on their engagement in struggles for social justice and citizenship.

Our arguments are based on qualitative research that has so far interviewed 111 women.^1^ The aim of the paper is to make visible the leadership these women exercise in populated areas. To that end, we chose to identify these women using *Snowball sampling* or *Snowball methodology* (32–34). The initial participants suggest new participants and so on (35,36). This is a non-probabilistic sampling technique that uses reference chains, in a kind of network (32,33,^37^).

The active search for female leaders in *favelas* was made through contacts, approaches, recommendations and participation in places where these women are found (civil society forums and community networks, among others) and where they are notably recognized for their socio-political performance, without delimitation of the study locus, considering that this search points us to interlocutors who come from all parts of the city of Rio de Janeiro. Semi-structured interviews were conducted through direct contact with participants, using an electronic questionnaire via mobile phone, all recorded in a database in the *Statistical Package for the Social Sciences* (SPSS). The research interviews began before the pandemic, with the aim of increasing the visibility of women’s leadership in popular areas, relating their experiences to the social determinants of health. As COVID-19 unfolded we resumed contact with research participants to better understand and make visible their leadership in the emerging pandemic context.

## Who are the other Marielles?

A black woman, daughter and mother, a woman from the *favela*, Marielle Franco was a sociologist, feminist, activist in defense of human rights and a Brazilian politician — a city councilor — who was assassinated in 2018. The questions about what motivated her murder and who ordered the crime are still awaiting answers.

A woman performing an active militant role, Marielle’s path intersects with those of many others and also with the other women referred to here. Her story emphasizes her social and political role, her connections with militancy for the guaranteeing of rights and her permanent struggle against various forms of segregation, violence, and oppression. So, Marielle is a symbol to the female leaders of the *favelas.*

The women represented here in our interviews narrate and portray a new form of political praxis. They transform their concerns into bridges (orchestrating and connecting different possibilities for action) and themselves and their collective into a virtuous movement for social change (38). To describe this process in the face of the COVID-19 pandemic, we will present their profile, portraying them as spokespersons who embody a sense of individual and collective struggle and resistance.

Our interlocutors are mostly black, corresponding to 90% of the interviewees; 80% are mothers; 44% completed a higher education diploma, 18% are currently attending higher education; 47% are married or live with a partner; and 65% are under 55. In Rio de Janeiro, several community-based organizations have emerged since the 1990s. They have been focused on the need for the social recognition of these women, since they represent the absolute majority in these places, and were set up as a result of the prominent demands for the mobilization of public resources to promote effective improvements in the living conditions within their territories. In this respect, 70% are linked to a community-based organization and 96% participate in collective activities (social movements, networks, collectives, and others). They work on several fronts, such as social assistance, health, education, culture, environment, among others, and it is important to note that 96% state that they work to promote community health. Networking (whether within the *favela* itself or outside it), participation in social movements, and the struggle for public policies that address needs — individual and collective — demonstrate the imperative visibility of what these women have been doing.

In view of the lack of coordinated action by the public authorities to confront COVID-19 in *favelas* and recognizing that social inequalities have a greater impact on mortality, we have sought to identify and monitor the set of strategies adopted by these women to deal with the pandemic. It is a time that calls for joint action to attend the urgencies of the vulnerable people who inhabit popular locations.

## The state of play: possible paths

In the context of the pandemic, the leaders — in full exercise of their micro-power — develop strategies to provide immediate responses to local emergencies. They mobilize different resources within civil society, activate public authorities, and map out different strategies to face the moment of crisis in public health. ‘We chase up, liaise, develop strategies. We can’t just cross our arms’, Rita from the Rocinha tells us.

We form a team, specifically to complement the actions: synthesis of laws related to the benefits and guidance on the flows and procedures to derive them; analysis of information revealing the fake news that so distresses the population; availability of people to perform market, pharmacy and food purchases, especially for the elderly and people with physical limitations; calculation and forwarding of various services performed online, with no charge deliveries; telephone contacts with the elderly emphasizing dialogue; provision of resources (food, hygiene products, wheelchairs, etc.). (Catia, from *favela* Barreira do Vasco)

Given the conditions imposed by the pandemic, with regard to the need for social isolation, resulting in loss of work and/or conditions for earning an income in the *favela* context, hunger is the foremost threat. Contributing to the food and nutritional security of families (especially those headed by women in conditions of extreme poverty and vulnerability) becomes a priority. Mothers in *favelas* are the people most affected, as many have had to leave their jobs to look after their own children. Vanessa, from the Buriti Congonhas *favela*, told us, ‘My first action involved two mothers who lost their jobs in the first week of the pandemic — one for being a day-worker and the other a street vendor. I needed to do something’. Due to their contacts and ability to talk to people inside and outside the *favelas*, all the women we are following began their efforts to cope with the demands arising from COVID-19 by acquiring baskets of basic grocery needs, along with hygiene and cleaning products. The strategies are diverse, ranging from support from individuals and companies and social media campaigns to birthday parties that transform the gift request into cash donations, so that they can subsidize the purchasing of essential goods. However, registering the neediest families and meeting their basic and immediate needs is not enough. The major concern over nutritional quality drives other action — campaigns carried out by several of these leaders have been launched so that they can obtain cash donations and thus purchase perishable foods such as eggs, vegetables, groceries, and fruits. Given that the basic-needs grocery baskets are rich in carbohydrates, it is also imperative to provide food products that protect the immune system, as pointed out by Pâmela, a leader in the Manguinhos *favela.* In this respect, the strengthening of this primary care reflects the role of health promotion for the sustainable well-being of the population.

The social and health conditions in the *favelas* challenge compliance with necessary prophylactic measures, such as hand and food hygiene. In many areas there is no basic sanitation and/or regular access to water. As a way of minimizing these difficulties, several campaigns have been carried out to install public sinks in alleys, so that people can at least access water, which is so essential to the strategy of preventing COVID-19. In addition to these objective strategies, these women coordinate actions with young volunteers so that, when delivering the basic-needs grocery baskets, guidance is provided on personal and environmental care.

Community communication is another tangible factor at this time. In view of the low educational level of the Brazilians from low-income classes, these female leaders have confirmed the need to facilitate the understanding of *favela* residents regarding the essential procedures for protecting individual and collective health. Thus, the communication needs to be deployed in a way that encourages people to adopt the necessary measures and follow all the standard procedures. So, empowering people and the community to act in accordance with the necessary guidelines requires empathy and trust, as confirmed by Amanda, from Maré. ‘Several strategies are being used for this purpose, such as placing banners, posters, spots with community radio stations, among other steps. It is necessary to speak the language of the *favela*’.

The *favela* breaks the rules for social distancing. The living space is generally shared by several people, making the street the extension of the house — their common space where interaction such as parties, fights, discussions, and leisure activities takes place. However, when people understand the dynamics of contagion and are encouraged in their efforts, they can feel more secure in facing the challenges posed by quarantine and social isolation measures.

Another challenge relates to commercial establishments inserted within the area of the *favela.* Due to the way they are set up, it is not practical to consider closing them. It is illusory to believe that small-scale entrepreneurs in places where the government is rarely present will adopt the criteria of other parts of the city. ‘This is a matter of day-to-day survival! Imagine whether the *favela* economy can stop?’ asks Lucia, from Complexo do Alemão. In the face of these specifics, it is essential to raise awareness about individual and, consequently, collective protection.

Considering the adversities to which people living in these popular locations are subjected, as well as their housing conditions, we are challenged to imagine a home with three or four children, all of whom are unable to go to school (or access online classes, since they usually have just a single mobile phone that is used by the whole family, with limited resources and often no internet access) and have to stay indoors for weeks. The proposed measures to ensure the continuity of education through virtual teaching platforms are not aimed at children and adolescents living in *favelas*. In addition to the challenges of shared space in the home and the lack of sufficient suitable equipment, the study routine requires the support of others who, in many cases, have not attended school — so this measure is not really feasible, due to the impossibility of it being implemented in the *favelas*, thus contributing to widening social inequities. Despite all this, the leaders are not bowed by the challenge and are in constant dialogue with the public authorities — acting in a network as a way of demanding action that takes into consideration the adversities presented by the moment.

The social and health conditions in the *favelas* challenge compliance with necessary prophylactic measures, such as hand and food hygiene. Julia, from Chatuba *favela*, says, ‘Not everyone has basic sanitation, a bathroom, or can go outside the small rooms in which three, four, or five people live. For these people to take all the recommended precautions is a challenge’. Accompanying the elderly and most vulnerable people has also been essential at this time, as the young leader Lays (21), a resident of the Palmeirinha *favela*, tells us:

One of our initial concerns was mental health. We, young people, have been challenged to pursue strategies. The first was to identify that they need to talk, to be heard. So, we started to visit them! (Lays, a resident of the Palmeirinha *favela*)

As the community activists are excellent orchestrators of networks inside the *favelas* and beyond, the management of volunteers in a chain of solidarity has been an important mechanism to support and give attention to the elderly. In addition, they provide care services to families who have lost their loved ones and are in dire need of emotional support. In this respect, several leaders are mediators between professionals and applied psychology services from universities that are providing voluntary care via the internet, contributing to the efforts towards controlling anxiety and other associated symptoms. In this sense, it is believed that promoting action to protect the health and well-being of the most vulnerable people is contributing to increasing equity, an aspect that is directly related to the principles of health promotion.

From this perspective of care, we must add violence against women. Due to inequalities in power relations, in times of pandemic, women’s vulnerability is even more exposed. In the face of this challenge, these leaders are sensitive and attentive and are of fundamental importance to this local protection network. In addition to taking care of battered women, they provide guidance on the system of guaranteed rights. Anazir, from Vila Aliança *favela*, says, ‘We formed a chain of goodness and started to take care of each other’.

In their daily activism, they build bridges and establish partnerships with health and social assistance units, a fundamental relationship for the care of victims of COVID-19. They are able to mediate access to services and provide assistance and guidance regarding the paths to be taken that guarantee their right to health and social assistance. So, an intersectorial approach is in the sights of collaborative action, since it involves coordinating public policies, civil society organizations, and the community.

Conscious of it being a case of ‘we for us’ and solidarity within the *favela* space, they promote health through understanding of the social determinations. The presence of public authorities in several *favelas* in Rio de Janeiro has been marked, in the context of the pandemic, only by the security policy. The so-called ‘operations’ against the ‘war on drugs’ — alluding to the armed control of popular locations by gangs — are in place, using practices that are fueled by class and racial division and urban segregation. The community activists presented here are not silent. In a movement marked by struggle and social resistance, they keep their activities going, assisting families and those who need support and to be heard. Their struggles are translated, in this pandemic, into recognition of citizenship, the restoring of core values that guide humanity, and the developing of strategies to overcome inequalities.

## Conclusion

The feminization of power is a growing phenomenon in contemporary society. However, we are interested in recognizing the participation of social activists and *favela* residents in this micro-political action involving mobilization and negotiation with different social agencies and the public authorities. These women, subject to agencies, are continually evolving repertoires to deal with the unequal structure to which they are permanently subjected. They recognize that the State does not act to deal with the conflicts caused by inequalities and consequently they seek ways to live better in *favelas*. The ability to connect and circulate within the city makes them autonomous and allows more freedom to build the paths that can lead them to resolving lives: their own, their families, and their communities — in the authentic sense that the term suggests: the place of belonging, neighborly relations, and solidarity.

The COVID-19 pandemic exposes the inequalities of Brazilian society and their consequences. Housing, sanitation, and access to health services are all offered to the poor, notably black, in a way that defines where the priorities in this class-based society lie, whose interests are based on individual desires, as opposed to collective interests. In this context, the strengthening of primary care and social and health services based on the community are essential to the role of health promotion and disease prevention.

Recognizing and ensuring social and cultural sustainability becomes a challenge to the current status quo in our society and a paradigm shift is essential — whereby competitiveness and individual interests must give way to the defense of solidarity.

The common intersectionality is an expression of gender, race, and class, associated with territorial factors, and is translated in this group as existence and resistance. The lived experience and different paths developed to deal with the pandemic reveal the importance of the struggle of these women. So, we seek in this study to identify and observe the set of strategies adopted by these women during the pandemic, confirming the important contribution offered by female leaders in the *favelas* to coping with the various problems that arose in the context of the COVID-19 pandemic. They were involved with housing, primary care, social and health services, nutritional support, community communication, and violence against women, among other local demands. It is essential to recognize their tactics, ability to organize and network, mobilization of the different resources of civil society, and development of a variety of strategies to face this moment of crisis in public health. They seek to create better conditions for the well-being of those who inhabit the *favelas*, where inequities critically expose the fundamental social distancing in Brazilian society.

## Notes

1 This research is approved by the Ethics Committee of the Catholic University of Rio de Janeiro (PUC-Rio).
